# Diosmin and Bromelain Stimulate Glutathione and Total Thiols Production in Red Blood Cells

**DOI:** 10.3390/molecules28052291

**Published:** 2023-03-01

**Authors:** Lukasz Gwozdzinski, Joanna Bernasinska-Slomczewska, Anna Wiktorowska-Owczarek, Edward Kowalczyk, Anna Pieniazek

**Affiliations:** 1 Department of Pharmacology and Toxicology, Medical University of Lodz, 90-752 Lodz, Poland; 2 Department of Oncobiology and Epigenetics, Faculty of Biology and Environmental Protection, University of Lodz, 90-236 Lodz, Poland

**Keywords:** diosmin, bromelain, red blood cells, oxidative stress

## Abstract

Diosmin and bromelain are bioactive compounds of plant origin with proven beneficial effects on the human cardiovascular system. We found that diosmin and bromelain slightly reduced total carbonyls levels and had no effect on TBARS levels, as well as slightly increased the total non-enzymatic antioxidant capacity in the RBCs at concentrations of 30 and 60 µg/mL. Diosmin and bromelain induced a significant increase in total thiols and glutathione in the RBCs. Examining the rheological properties of RBCs, we found that both compounds slightly reduce the internal viscosity of the RBCs. Using the MSL (maleimide spin label), we revealed that higher concentrations of bromelain led to a significant decrease in the mobility of this spin label attached to cytosolic thiols in the RBCs, as well as attached to hemoglobin at a higher concentration of diosmin, and for both concentrations of bromelain. Both compounds tended to decrease the cell membrane fluidity in the subsurface area, but not in the deeper regions. An increase in the glutathione concentration and the total level of thiol compounds promotes the protection of the RBCs against oxidative stress, suggesting that both compounds have a stabilizing effect on the cell membrane and improve the rheological properties of the RBCs.

## 1. Introduction

Due to their healing properties, variety of compounds of plant origin are used in the treatment of cardiovascular diseases. This wide group includes diosmin, a flavone glycoside found abundantly in citrus fruits. Recently, numerous in vitro and in vivo studies have demonstrated the wide range of biological activity of diosmin, which includes antioxidant, antihyperglycemic, anti-inflammatory, anti-mutagenic, and anti-ulcer properties, as well as anti-cancer and antibacterial properties. This flavonoid is employed in the treatment of cardiovascular diseases, particularly chronic venous insufficiency, such as varicose veins, as well as to improve microcirculation. Diosmin is also used to improve liver cells protection and for neuroprotection [1,2]. However, this flavonoid’s molecular mechanism of action has not yet been elucidated, although some potential molecular targets for diosmin have been reported, e.g., P-glycoprotein (P-gp), IKKβ, acetylcholinesterase (AChE), and aldose reductase (AR) [3]. Nevertheless, further research expounding diosmin’s mechanism of action in cellular processes is still needed.

Many studies have demonstrated the antioxidant properties of diosmin, including effective modulation of the activity of a number of enzymes and biomarkers, which were related to the disturbance of the oxidative balance in various diseases. Oxidative imbalance, which results in oxidative stress, is associated with the development of numerous diseases, including cardiovascular diseases, e.g., myocardial ischemia, varicose vein, thrombosis, stroke (ischemia-reperfusion damage), but also diabetes, neurodegenerative diseases, cancer, and many others [4,5,6,7]. As a drug, diosmin is often used with other flavonoids. It has been shown that a combination of diosmin with hesperidin was very effective in the treatment of chronic venous insufficiency [8]. Diosmin showed antioxidant properties by reducing the oxidative stress in the rat heart following in vivo ischemia/reperfusion [9]. The ischemia-reperfusion injury was characterized by a decrease in the activity of enzymatic antioxidants (SOD, CAT, and GPx) and a decrease in the level of GSH [10]. The administration of diosmin led to an increase in enzymatic antioxidants and the level of GSH. A decrease in lipid peroxidation products was also found [9].

Another types of diosmin activity in oxygen metabolism can be observed in intracellular processes of the selected cell lines. It has been shown that in DU145 cells, diosmin led to radical oxidative stress by increasing the overall production of reactive oxygen species (ROS), which caused changes in the potential of the mitochondrial membrane and, as a consequence, apoptosis and cell death [11]. Diosmin also initiated genotoxicity by inducing double-strand breaks in DNA and creating micronuclei [12]. In MCF-7 and MDA-MB-231 breast cancer cells, diosmin initiated an increase in the p53 protein levels. A similar increase in the level of p27 protein was observed in MDA-MB-231 and SK-BR-3 cells. All three cell lines have also been noted to increase the p2 protein levels. Moreover, in MCF-7 cells, this flavonoid led to the externalization of phosphatidylserine, an increase in the activity of multiple caspases, and the depolarization of the mitochondrial membrane potential. Diosmin also induced the oxidative stress in the studied cell lines. The highest increases in ROS, total superoxide, and mitochondrial superoxide production were observed in the MCF-7 cells. Moreover, in these cells, diosmin initiated single- and double-strand DNA breaks. The flavonoid also initiated autophagy in all cell lines tested. This process was enhanced in MDA-MB-231 and SK-BR-3, but not in MCF-7 cells [13].

Pineapple fruit and the other pineapple plant parts, such as leaves and stem, were formerly successfully used in folk medicine to treat open wounds and inflammation. The studies of the pineapple extract revealed a number of interesting biological properties, such as bactericidal, anti-swelling, and anti-inflammatory effects [14]. Bromelain, a mixture of protein digesting enzymes, such as thiol endopeptidases and phosphatase, glucosidase, peroxidase, cellulase, escharase, and several protease inhibitors, is the main ingredient of pineapple extract. Bromelain has cardioprotective, immunomodulating, and antioxidant properties. This enzyme is used in the treatment of angina, bronchitis, and sinusitis, but also in thrombophlebitis. In addition, bromelain also has some anti-cancer effects and promotes cancer cells apoptosis [15,16]. In vivo and in vitro studies have shown that bromelain can reduce the symptoms of cardiovascular diseases [17,18]. Moreover, it has anticoagulant and fibrinolytic properties which have been used in the treatment of thromboembolism, as well as in the treatment of thrombophlebitis. It has been shown that bromelain initiates thrombus destruction, as well as reduces platelet clumping and lowers blood viscosity [19,20]. It was also found in vivo that bromelain’s fibrinolytic properties were effective in dissolving atherosclerotic plaque, reducing the risk of developing atherosclerotic disease. Due to its anti-inflammatory properties, bromelain reduces postoperative symptoms in patients undergoing alveolar ridge preservation after tooth extraction [21].

Oxidative stress can lead to changes in the activity of antioxidant systems in erythrocytes, damaging in their cell membrane, and changing their rheological properties. Consequently, such actions will cause obvious changes in the function of red blood cells. In the light of recent findings, erythrocytes seem to be an important regulator of cardiovascular function in pathophysiological conditions. The RBCs are critically involved in cardiovascular homeostasis as a regulator of cardiovascular function by exporting adenosine triphosphate (ATP) and nitric oxide (NO) bioactivity and in redox balance via their effective antioxidant systems [22,23]. The alteration in the function of the RBC may lead to oxidative stress, alterations in protein content and enzyme activities, and increased adhesion to the vascular wall. Such disorders, defined as ‘erythropathy,’ ultimately result in the ability to trigger impairment of vascular and cardiac function [24].

The aim of this research was to study the effect of diosmin and bromelain on human red blood cells. The influence of both compounds on the induction of the oxidative stress and the total antioxidant capacity of the RBCs was determined. Moreover, the level of total thiols and glutathione, as well as the level of amino groups in the hemolysate, were specified. The internal viscosity of the RBCs and the conformational state of hemoglobin were examined. The fluidity of the RBCs plasma membranes was estimated after treatment with diosmin and bromelain.

## 2. Results

In this work, the influence of diosmin and bromelain on the initiation of the oxidative stress in red blood cells was investigated. The red blood cells from healthy individuals were treated with two concentrations of diosmin and bromelain at a final concentration of 30 µg/mL and 60 µg/mL. Our previous studies on endothelial cells showed that, at a concentration of 50 µg/mL, these compounds cause a decrease in cell survival by about 7–15% (unpublished data). Hence, the above choice of concentrations of the tested compounds.

**Figure 1:**
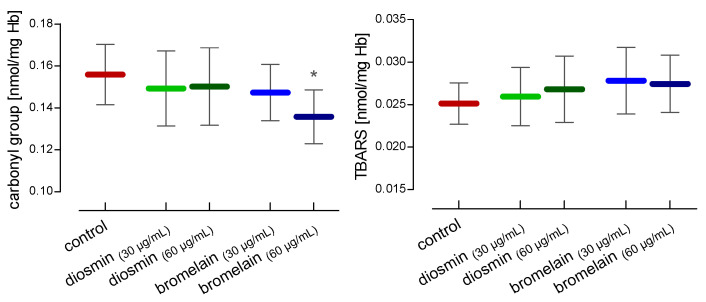
Alterations in carbonyl group and TBARS concentrations in RBCs after 24 h of incubation with diosmin or bromelain. The data were expressed as mean ±standard deviation, n = 16. Statistical significance: * *p* < 0.05 vs. control.

Figure 1 shows the level of TBARS and carbonyl compounds after RBC treatment with diosmin and bromelain. We found that both compounds lowered the total level of carbonyl compounds, but the results were not statistically significant. A significant decrease in the level of carbonyl groups was observed only in RBCs treated with bromelain at a concentration of 60 µg/mL. However, the TBARS level remained unchanged.

**Figure 2:**
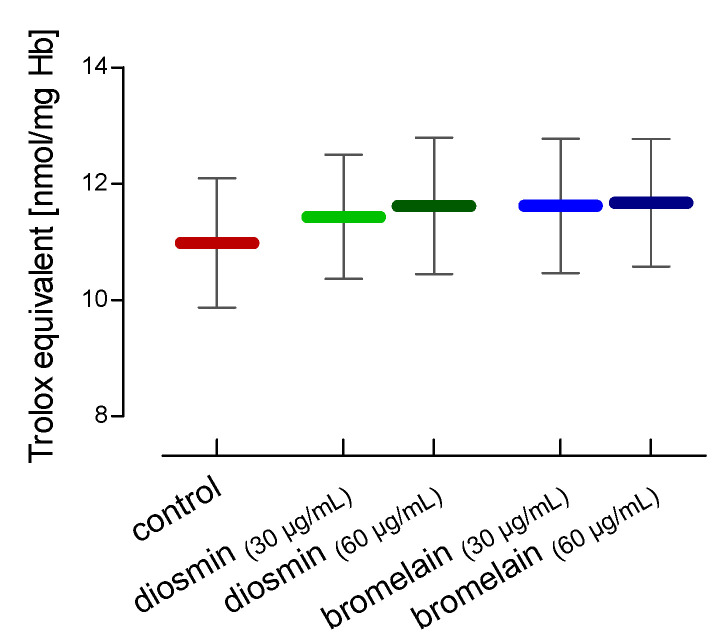
Alterations in the total non-enzymatic antioxidant capacity in RBCs after 24 h of incubation with diosmin or bromelain. The data were expressed as mean ± standard deviation, n = 16.

The total non-enzymatic antioxidant capacity (NEAC) was also determined. Diosmin and bromelain led to an increase in the total non-enzymatic antioxidant capacity, but these results were not statistically significant (Figure 2).

**Figure 3:**
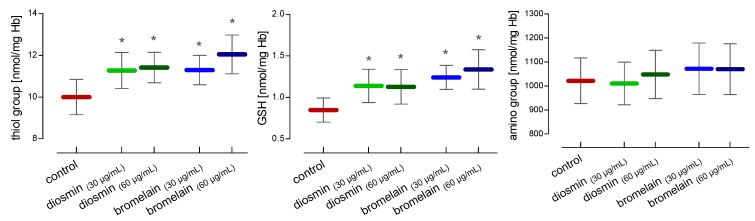
Alterations in thiol and amino groups and glutathione concentrations in RBCs after 24 h of incubation with diosmin or bromelain. Data were expressed as mean ±standard deviation, n = 16. Statistical significance: * *p* < 0.05 vs. control.

In the hemolysates of the RBCs treated with diosmin and bromelain, the total level of thiols, glutathione, and amino groups was determined. In our study diosmin and bromelain led to a significant increase in total thiols and glutathione in red blood cells. However, no differences were found in the level of amino groups (Figure 3).

**Figure 4:**
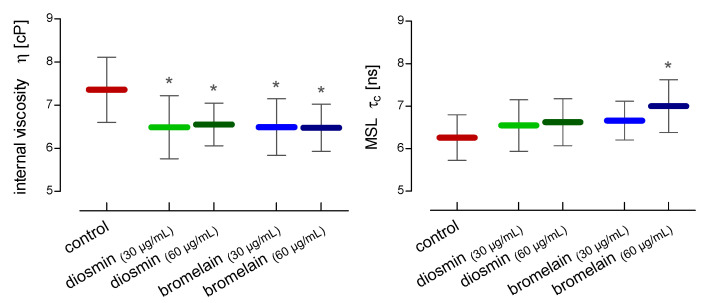
Alterations in internal viscosity and maleimide (MSL) spin label mobility in RBCs after 24 h of incubation with diosmin or bromelain. The data were expressed as mean ± standard deviation, n = 12. Statistical significance: * *p* < 0.05 vs. control.

The influence of both compounds on the internal viscosity of the red blood cells was investigated using tempamine. The condition of the internal proteins was determined using MSL. The conducted research showed that diosmin and bromelain led to a statistically significant decrease in the internal viscosity of the cells (Figure 4). One of the objectives of the study was to investigate the effect of diosmin and bromelain on the internal components of red blood cells, mainly hemoglobin, which accounts for over 95% of all proteins present inside the RBCs. MSL spin label rotation allows for the evaluation of the changes in the RBCs internal environment. From the EPR spectra of the whole RBCs labeled with MSL, the relative rotational correlation time (*τ_c_*) was calculated. Figure 4 shows the effects of diosmin and bromelain on the mobility of proteins and peptides in the whole red blood cells. A slight increase in *τ_c_* upon diosmin and bromelain RBCs treatment was observed. Nevertheless, a higher bromelain concentration (60 µg/mL) caused a significant increase in this parameter in the RBCs.

**Figure 5:**
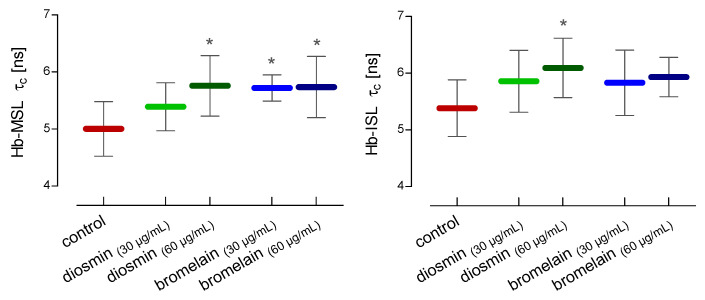
Alterations in the rotational correlation time of MSL and ISL attached to the crude hemoglobin after 24 h of incubation of RBCs with diosmin or bromelain. The data were expressed as mean ± standard deviation, n = 11. Statistical significance: * *p* < 0.05 vs. control.

For the estimation of the changes in the conformational state of hemoglobin in the hemolysate, two spin labels, MSL and ISL, were used. After the RBCs treatment with a higher concentration of diosmin (60 µg/mL) and both concentrations of bromelain (30 µg/mL and 60 µg/mL), compared to the control, a statistically significant increase in the rotational correlation time of the MSL spin label attached to hemolysate proteins was observed (Figure 5). In the case of the ISL spin label bound to hemolysate proteins, a statistically significant increase in rotational correlation time compared to the control was observed only after the incubation of the RBCs with a higher concentration of diosmin (60 µg/mL) (Figure 5).

**Figure 6:**
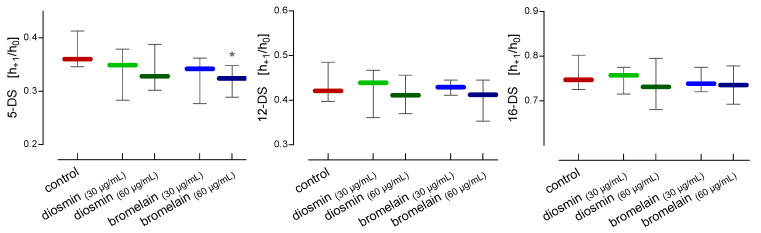
Alterations in the *h*_+1_/*h*_0_ parameters of 5-, 12-, and 16-doxy-stearic acid incorporated into the erythrocyte’s lipid membranes after 24 h of incubation with diosmin or bromelain. The data were expressed as the median with an interquartile range, n = 11. Statistical significance: * *p* < 0.05 vs. control.

The fluidity of the red blood cell membrane was assessed using three spin-labeled fatty acids 5-DS, 12-DS, and 16-DS. These acids have a paramagnetic nitroxide group located at different distances from the carboxyl group of the fatty acid chain, so they enable the determination of the fluidity of the lipids at different depths of the lipid monolayer of the cell membrane. Comparing the values of the *h*_+1_/*h*_0_ (the ratio of the height of the low-field line amplitude to the height of the midfield line amplitude) parameter, determined with the EPR spectra of the spin label incorporated into the membranes, we showed differences in the fluidity of RBCs cell membranes treated with diosmin and bromelain (Figure 6). The decrease in the *h*_+1_/*h*_0_ parameter of the 5-DS after the RBCs treatment with diosmin or bromelain reflects the slight decrease in lipid fluidity in the subsurface region of the membrane. A statistically significant decrease in membrane fluidity near the surface of the membrane lipid monolayer was observed only after the incubation of RBCs with 60 µg/mL of bromelain. On the other hand, we found no changes in the lipid fluidity in the deeper regions of the monolayer after the RBCs treatment with diosmin and bromelain using labeled 12-DS and 16-DS fatty acids.

## 3. Discussion

Diosmin (diosmetin 7-O-rutinoside) is a disaccharide derivative which consists of aglycone diosmetin. After the administration of diosmin into the digestive system, enzymes of the intestinal microflora hydrolyze diosmin to its aglycone, diosmetin [2]. Moreover, bromelain is a mixture of protein digesting enzymes. Interestingly, bromelain is largely absorbed in the body, without losing proteolytic activity, and it does not cause any major side effects [15]. Many studies have shown that diosmin and bromelain can initiate ROS production and oxidative stress in cells [8,12,25,26]. Furthermore, it has been shown that bromelain can initiate ROS-induced ferroptosis in Kras mutant CRC cells via ACSL-4. ACSL-4 performs a key role in regulating ferroptosis and directs cells to this type of cell death [27]. Bromelain and diosmin reduced the viability of tumor cells by inducing apoptosis via the mitochondrial pathway [28,29].

The influence of both compounds on the initiation of oxidative stress was investigated. We found that both compounds lowered the total level of carbonyl compounds. The TBARS level remained unchanged; however, the tendency to increase non-enzymatic antioxidant capacity was observed. In our previous paper, we showed a decrease in NEAC in plasma, as well as a decrease in the level of thiols in plasma and hemolysate obtained from varicose vein RBCs. On the other hand, we observed an increase in parameters related to oxidative stress, such as the level of protein carbonyl compounds and the level of thiobarbituric acid reactive substances (TBARS) in RBCs from varicose veins [30].

Erythrocytes transport oxygen to cells and tissues, but are also exposed to oxygen reactive forms, both in the circulation and within the cell by the self-oxidation of hemoglobin [31,32]. Therefore, the RBCs have the specialized antioxidant enzyme systems, such as superoxide dismutase, catalase, and thioredoxin peroxidase, and low molecular weight antioxidants, such as glutathione, ascorbic acid, tocopherol, and others [33]. Glutathione is the most important low molecular weight antioxidant present in RBCs, and its concentration (0.6–3.6 mM) is higher compared to other thiols and other antioxidants, such as ascorbic acid or tocopherol [34,35]. The measurement of glutathione (GSH) and thiol content in RBCs can be used as a marker of oxidative stress. Thiol-containing compounds perform an important role in protecting the biological systems from oxidative damage. Because thiol compounds are very sensitive to oxidation, they can be oxidized by mild oxidants, such as superoxide and hydrogen peroxide. Therefore, they are crucial in protecting cells from ROS [36]. The total level of thiols, glutathione, and amino groups was determined. Diosmin and bromelain induced a statistically significant increase in total thiols and glutathione at both concentrations used. The RBCs have a specialized enzyme system for the synthesis of glutathione, consisting of glutathione synthase, glutamate-cysteine ligase-catalytic subunit, and glutathione reductase. In turn, oxidized glutathione and glutathione conjugates formed under various conditions are expelled from the red blood cells. Sustainable exports of reduced glutathione and other thiols, such as cysteine and homocysteine, from the RBCs to plasma have also been demonstrated [37]. These results indicate that red blood cells can significantly affect the extracellular pool of glutathione, participating in cooperation with the liver and other tissues in the synthesis and metabolism of GSH. In addition, it has been shown that the ability of the RBCs to synthesize glutathione is 150 times higher than the glutathione turnover rate. These results indicate a significant reserve of efficiency of the RBCs for the synthesis of glutathione [38].

We have determined the red blood cells parameters that affect the rheological properties and the deformability of the RBCs as they pass through the capillaries in microcirculation. The deformability of the RBCs is influenced by factors such as interior viscosity, the fluidity of plasma membranes, and the condition of the membrane cytoskeleton [39]. We specified the effect of diosmin and bromelain on the internal viscosity of red blood cells using EPR spectroscopy with tempamine. Both compounds led to a significant decrease in the viscosity of the intracellular fluid of the RBCs.

Changes in the viscosity of the internal fluid prompted us to study the effect of diosmin and bromelain on the internal components of red blood cells, mainly hemoglobin, which constitutes over 95% of all proteins present in the RBCs. The use of the spin labeling method in EPR spectroscopy made it possible to determine the changes in the mobility of internal components (cytosolic thiols), including hemoglobin. The mobility of cytoplasmic peptides and proteins in the whole red blood cells was investigated using the MSL. The label crosses the RBCs membrane and reacts with the thiol groups of peptides and proteins. Our earlier studies showed that over 90% of the spin label binds to components present in the cytosol, mainly to glutathione, and to a lesser extent, to hemoglobin and membrane proteins [40]. The treatment of red blood cells with diosmin and bromelain led to an increase in the relative rotational correlation time of the bound to the cytosolic thiols. However, a statistically significant increase in *τ_c_* was only observed with a higher concentration of bromelain.

One of the objectives of the study was to investigate the effect of diosmin and bromelain on the internal components of red blood cells, mainly hemoglobin, which accounts for over 95% of all proteins present inside the RBCs. Potential changes in the conformational state of hemoglobin were determined using two labels, MSL and ISL, reacting with the thiol groups. Both spin labels have been shown to bind to the –SH groups of the cysteine-93 of the β-globin chains in hemoglobin [41,42]. The RBCs treatment with diosmin and bromelain resulted in an increase in the rotational correlation time of the bound spin label to hemoglobin. This increase of *τ_c_* of MSL was statistically significant for the higher concentration of diosmin and both concentrations of bromelain used. An increase in *τ_c_* for both compounds was also observed for the ISL, howeverthese results were statistically significant for higher concentration of diosmin.

The fluidity, microviscosity, or stiffness (rigidity) of the plasma membrane is a frequently used parameter in determining its physical state. Membrane fluidity depends on a number of factors, such as the chemical structure of phospholipids, the degree of saturation with fatty acids, the ratio of protein to lipids, and the presence of cholesterol. Plasma membranes are characterized by a fluidity gradient from the water boundary to the inside of the bilayer [43,44]. Membrane fluidity determines the passive and active transport of electrolytes and non-electrolytes across membranes into and out of the cell [45,46]. In addition, the fluidity of the membrane affects the deformability of the red blood cells when passing through capillaries with a diameter smaller than the diameter of the RBCs.

Treatment of red blood cells with diosmin and bromelain led to a decrease in lipid fluidity in the subsurface membrane compared to the control, as revealed by the 5-DS probe. However, these results were statistically significant for higher concentration of bromelain. We did not observe changes in lipid fluidity in the deeper regions of the monolayer (hydrophobic core) using 12-DS and 16-DS labeled fatty acids and treating the RBCs with diosmin and bromelain. Using three fluorescent probes, 6-AS, 12-AS, and 16-AP, derivatives of stearic and palmitic acids, respectively, located at different depths of the monolayer vesicles, it was shown that naringenin, rutin, genistein, genistin, biochanin A, equol, dihydrodaidzein, and dihydrogenistein led to a decrease in membrane fluidity. The results of the study suggest that both glycosides and aglycones acted similarly to cholesterol and α-tocopherol, which are located in the hydrophobic core of the membrane, and led to a strong decrease in lipid fluidity in this region of the membrane [47]. In addition, flavonoids have the ability to stabilize membranes by reducing their fluidity [47]. In turn, orally administered diosmin led to an increase in the RBCs stiffness and a decrease in cholesterol in red blood cell membranes in rats. However, there were no changes in the osmotic fragility of RBCs, but a dose-dependent decrease in the ratio of cholesterol to phospholipids was found [48].

## 4. Material and Methods

### 4.1. Chemicals

The following chemicals were purchased from Sigma Chemical Co. (St. Louis, MO, USA): 4-Amino-TEMPO (tempamine), 4-Maleimido-TEMPO (MSL), 4-(2-Iodoacetamido)-TEMPO (ISL), 5-doxyl-stearic acid (5-DS), 12-doxyl-stearic acid (12-DS), 16-doxylstearic acid (16-DS), o-phthalaldehyde (OPA), 4,4-dithiodipyridine, 2,4,6-trinitrobenzene sulfonic acid (TNBS), 2,4-dinitrophenylhydrazine (DNPH), and 2,4,6-tripyridyl-S-triazine (TPTZ). All other reagents of analytical purity were obtained from POCH S.A. (Gliwice, Poland). The investigated compounds, diosmin (3′,5,7-Trihydroxy-4′-methoxyflavone 7-rutinoside) and bromelain from the pineapple stem, were purchased from Sigma-Aldrich (St. Louis, MO, USA). Both compounds were dissolved according to the manufacturers’ suggestions: diosmin was dissolved in DMSO, and bromelain was dissolved in PBS.

### 4.2. Red Blood Cells Isolation

All experiments were performed on human erythrocytes isolated from the buffy coat obtained from the Regional Center for Blood Donation and Hemotherapy in Lodz. For the RBC separation, the blood buffy coat was washed three times with PBS (10 mM phosphate-buffered saline, pH 7.4). Erythrocytes were suspended in Ringer’s solution to a hematocrit of 50% and separately incubated for 24 h at 37 °C with diosmin at a final concentration of 30 µg/mL and 60 µg/mL or bromelain at a final concentration of 30 µg/mL and 60 µg/mL. After incubation, the cells were washed with PBS and used for future experiments.

EPR measurements of the RBCs’ internal viscosity, membrane fluidity, internal peptides, and proteins changes were conducted on the whole RBCs. The remaining experiments were carried out on hemolysate. Every single experiment was performed on cells or hemolysate from one donor, and n-numbers represent cells from different individuals.

### 4.3. Hemolysate Preparation

The hemolysate was obtained from washed RBCs by adding cold water at a ratio of 1:1 and vortexed for 10 min, according to the method described by Drabkin [49]. The hemolysate was centrifuged at 4000× *g* for 10 min to separate erythrocyte ghosts. The total hemoglobin (Hb) concentration in the hemolysate was estimated as cyanmethemoglobin using Drabkin’s reagent, and absorbance was measured at 546 nm [49]. The molar absorption coefficient of hemoglobin was used to calculate the protein concentration in the samples (ε = 44 mmol^−1^·L·cm^−1^).

### 4.4. Determination of Carbonyl Groups

The protein carbonyl content in hemolysate was determined with 2,4- dinitrophenylhydrazine (DNPH) [50]. The reaction between the protein carbonyl groups and DNPH led to the formation of protein-conjugated dinitrophenylhydrazones (DNP), of which the absorbance can be measured at 370 nm. The content of the carbonyl compounds was calculated using the millimolar absorption coefficient (ε = 22 mmol^−1^·L·cm^−1^) and expressed as nanomoles per milligram of hemoglobin (nmol/mg Hb). The data were expressed as the mean ± standard deviation (n = 16).

### 4.5. Determination of Thiobarbituric Reactive Substances

The lipid peroxidation in the hemolysate was measured by determining the interaction of thiobarbituric acid (TBA) with the breakdown product of lipid peroxidation under acid pH conditions using TBARS assay [51], with the modifications of Rice-Evans et al. [52]. The end product of the reaction was determined at 535 nm, and the TBARS concentration was calculated using the millimolar absorption coefficient (ε = 156 mmol^−1^ L·cm^−1^) and expressed as nanomoles per milligram of hemoglobin (nmol/mg Hb). The data were expressed as the mean ± standard deviation (n = 16).

### 4.6. Determination of Thiol Groups

The concentration of the thiol groups in the hemolysate was determined using the Egwim and Gruber method with 4, 4′-dithiodipyridine [53]. The absorbance of 2-thiopyridon, a product of the reaction between thiols and 4, 4′-dithiodipyridine, was measured at 324 nm. The concentration of the thiol group was estimated based on the standard curve prepared from various concentrations of reduced glutathione and expressed as nanomoles per milligram of hemoglobin (nmol/mg Hb). The data were expressed as the mean ± standard deviation (n = 16).

### 4.7. Determination of Glutathione Content

The concentration of reduced glutathione (GSH) in the hemolysate was estimated according to the fluorescence assay using o-phthalaldehyde (OPA) [54]. The reaction of GSH with o-phthalaldehyde led to a fluorescent product with high fluorescence, allowing GSH to be precisely quantified. The OPA-derived fluorescence was measured at an excitation wavelength of 365 nm and emission at 430 nm. The glutathione concentration was calculated using the calibration curve prepared from various concentrations of reduced glutathione and expressed as nanomoles per milligram of hemoglobin (nmol/mg Hb). The data were expressed as the mean ± standard deviation (n = 16).

### 4.8. Determination of Amino Groups

The concentration of the free amino group in hemolysate was estimated using the method described by Crowell et al. [55]. The absorbance of the product of the reaction between amines and 2,4,6-trinitrobenzene sulfonic acid (TNBS), was measured at 335 nm. The concentration of the amino groups was estimated based on the standard curve prepared for different homocysteine concentrations, and was calculated as nmol/mg per milligram of hemoglobin (nmol/mg Hb). The data were expressed as the mean ± standard deviation (n = 16).

### 4.9. Total Non-Enzymatic Antioxidant Capacity

The non-enzymatic antioxidant capacity (NEAC) of the hemolysate was determined by the reaction involving the reduction of Fe^3+^-TPTZ (iron[III]-2,4,6-tripyridyl-S-triazine) to Fe^2+^ TPTZ in presence of antioxidants. The absorbance of the reaction product, measured at 593 nm, is proportional to the level of antioxidants [56]. A calibration curve was prepared using different concentrations of Trolox. The results were calculated as nmol of Trolox equivalents per milligram of hemoglobin. (nmol/mg Hb). The data were expressed as the mean ± standard deviation (n = 16).

### 4.10. Electron Paramagnetic Resonance (EPR)

The EPR spectra were estimated on the Bruker ESP 300 E spectrometer (Rheinstetten, Germany) at room temperature, operating at a microwave frequency of 9.73 GHz. The instrumental settings were as follows: the microwave power was 10 mW, the center field was set at 3480 G, with a 100 kHz modulation frequency and a range of 80 G, a modulation amplitude of 1.01 G, and a time scan of 256 s.

### 4.11. Internal Viscosity of RBCs

The internal viscosity of the RBCs was estimated using tempamine, according to the method described by Morse [57]. The RBCs (hematocrit: 50%) were labeled with tempamine dissolved in ethanol solution (the final concentration of tempamine was 1 mM) for 0.5 h at room temperature. For the elimination of the extracellular signal of the label, before the measurement, the RBCs were washed with 80 mM potassium ferricyanide in 5 mM phosphate buffer pH 7.4. From the EPR spectra, the relative rotational correlation time (*τ_c_*) was calculated from an equation formulated by Keith et al. [58];

(1) $$\tau_{c}=kw_{0}\sqrt{\frac{h_{0}}{h_{-1}}}-1$$

where *τ*_𝑐_ is the time when the spin label undergoes a full rotation, 𝑘 is constantly equal to 6.5 × 10^−10^ [s], *w*_0_ is the width of the midline of the spectrum, *h*_0_ is the height of the midline of the spectrum, and *h*_−1_ is the height of the high-field line the of the spectrum.

The erythrocyte internal viscosity was calculated according to the following formula:

(2) $$\eta_{\left(RBC\right)}=\frac{\tau_{c\;\left(RBC\right)}}{\tau_{c\;\left(H_{2}O\right)}}\eta_{\left(H_{2}O\right)}$$

where; *τ_c_*_(*RBC*)_ is the rotational correlation time for tempamine inside the erythrocyte, *τ_c_*_(*H*_2_*O*)_ is the rotational correlation time for tempamine in water, and *η*_*H*_2_*O*_ is the water viscosity equal to 1 cP. The data were expressed as the mean ± standard deviation (n = 12).

### 4.12. The RBCs Internal Peptides and Proteins Changes

For the analysis of the changes of internal cytoplasmic peptides and proteins of erythrocyte EPR, 4-Maleimido-TEMPO (MSL) was used as the spin label [40]. The RBCs (hematocrit: 50%) were labeled with MSL in an ethanol solution (the final concentration of MSL was 1 mM) for 1 h at room temperature. For the elimination of the spin label excess, the RBCs were washed several times with cold phosphate buffer saline (PBS), until the disappearance of the EPR signal in the supernatant. From the EPR spectra, the relative rotational correlation time (*τ_c_*) was calculated from an equation formulated by Keith et al. [58]. The data were expressed as the mean ± standard deviation (n = 12).

### 4.13. The Conformational State of Hemolysate Proteins

For the investigation of the conformational changes of hemolysate proteins (mainly hemoglobin), two spin labels, MSL and ISL, were used. Under physiological conditions, both spin labels reacted with the thiol groups of proteins [59] The hemolysate was labeled using ethanol solutions of MSL or ISL and incubated for 1 h at 4 °C (the final concentration of the spin label was 0.1 mM). The unbound spin label was removed by 24 h of dialysis against 10 mM phosphate buffer (pH = 7.4). From the obtained EPR spectra, the mobility of the spin label attached to the proteins was estimated by calculating the rotational correlation time (*τ_c_*) [58]. The data were expressed as the mean ± standard deviation (n = 11).

### 4.14. RBCs Membrane Fluidity

The lipid membrane fluidity of the RBCs was evaluated with the EPR technique using spin-labeled fatty acids (5-DS, 12-DS, and 16-DS).

An ethanol solution of the spin label fatty acid was added into the RBCs (hematocrit: 50%) to the final concentration of 0.1 μM and incubated for 30 min at room temperature. From the EPR spectra, the *h*_+1_/*h*_0_ parameter was calculated, where *h*_+1_ is the height of the low-field line of the spectrum, and *h*_0_ is the height of the mid-line of the spectrum. The data for 5-DS, 12-DS, and 16-DS were expressed as the median and interquartile range (IQR: from lower quartile Q1 to upper quartile Q3) (n = 11).

### 4.15. Statistical Analysis

The normality of data was tested using the Shapiro–Wilk test, and variance homogeneity was verified with the Levene’s test. For the variables showing a departure from normality, the data were presented as the median and interquartile range (IQR: from lower quartile Q1 to upper quartile Q3). For variables data, with no departure from normality, data were presented as mean ± standard deviation (SD). The significance of the differences between the groups was estimated by a one-way ANOVA using Tukey’s post hoc multiple comparisons test for the data with no departure from normality. For the data with a departure from normality, the non-parametric Kruskal–Wallis test was used. Statistical significance was accepted at *p* < 0.05. The statistical analysis was performed using Statistica v. 13.3 (StatSoft Polska, Krakow, Poland).

## 5. Conclusions

Diosmin and bromelain have different antioxidant effects on human red blood cells. We found an increase in the concentration of glutathione in the cell and in the total level of thiol compounds, which contributes to the antioxidant protection of the RBCs and plasma against oxidative stress. Both compounds have a stabilizing effect on the RBCs cell membrane and show a tendency to decrease the viscosity of the cell interior and decrease the fluidity of the cell membrane, which improves the rheological properties of the RBCs. These results partly explain the beneficial effects of diosmin and bromelain in the treatment of cardiovascular diseases and might help to protect RBCs against erythropathy.

## Data Availability

The data presented in this study are available on request from the corresponding author.

## Abbreviations

RBCs	erythrocytes
EPR	electron paramagnetic resonance
TBARS	thiobarbituric acid reactive substances
GSH	glutathione
NEAC	non-enzymatic antioxidant capacity
SOD	superoxide dismutase
CAT	catalase
GPx	glutathione peroxidase
ROS	reactive oxygen species
MSL	4-Maleimido-TEMPO
ISL	4-(2-Iodoacetamido)-TEMPO
5-DS	5-doxy-stearic acid
12-DS	12-doxy-stearic acid
16-DS	16-doxy-stearic acid
